# A Discussion of Conflicts of Interest in Plastic Surgery and Possible Remedies

**DOI:** 10.1097/GOX.0000000000002043

**Published:** 2018-12-12

**Authors:** Eric Swanson, Tim Brown

**Affiliations:** * Private Practice, Leawood, Kans.; † Private Practice, Berwick, Victoria, Australia.

## THE PROBLEM

Conflict of interest (COI) is a subject of intense interest, to the extent that an entire issue of the *Journal of the American Medical Association* was devoted to this subject last year.^1^ In 2014, the American Society of Plastic Surgeons created a task force to address this problem.^2^ Conflict of interest was the lead story of a recent issue of *Plastic Surgery News*.^2^ Remarkably, about half of U.S. physicians, and 61% of surgeons, received payments from the pharmaceutical and medical device industries in 2015, amounting to $2.4 billion, including 136 plastic surgeons who received >$10,000 each.^3^

Before the early 1980s, there was little intersection between medicine and industry. Collaboration between the medical profession and the corporate world has increased.^4^ The link between commercial funding and study conclusions is undeniable in plastic surgery.^5,6^ When industry-supported Continuing Medical Education programs are conducted at resort hotels and upscale restaurants, the boundary between education and industry marketing is blurred.^7^

At meetings, plastic surgeons often declare, “I have no relevant conflict of interest” or “I have no conflicts that would affect the content of my presentation.” Luce laments that sometimes the duration of the disclosure slide presentation could be measured in nanoseconds, as reported in Plastic Surgery News.^2^ The speaker usually decides whether a conflict is relevant. Presenters sometimes comment, wryly, “I have no conflict of interest, unfortunately,” recognizing, and trivializing, the financial benefit of a COI. Some speakers display a long list of conflicts and suggest that because they have so many, they are at least “equal opportunity conflicters.” Some investigators believe that if they previously received money but no longer receive payments, they are no longer conflicted. Is there an expiry date for financial conflicts? Although some journals specify a 3-year period before submission, full disclosure is preferred, allowing the reader to decide on the merits.^8^

## DEFINITION

Luce^6^ defines COI: “Conflicts in ethically problematic situations are those in which the practitioner participates in clinical investigation of new devices/technology, publishes that experience, and, in parallel, is paid a consultant’s fee by the manufacturer.” Fineberg^9^ believes that a COI exists when a reasonable person would interpret the financial circumstances as sufficient to influence a physician’s judgment. When the reasonable person standard is used, the “appearance of” a COI is redundant.^9^ Proof of patient harm is not a requisite for COI; there are no “potential” COIs.^10^

## INDUSTRY PAYMENTS TO INDIVIDUALS

The American Society of Plastic Surgeons recently introduced dollar ranges for reporting financial conflicts.^2^ However, no data are available regarding a monetary threshold for a COI.^11^ Using Open Payments data, a 2016 study found that receipt of industry-sponsored meals, even just a single meal, was associated with an increase in the rate of prescribing the promoted brand-name drug.^12^ The more money doctors receive, on average, the more brand-name medications they prescribe.^13^ The evidence shows that even small gifts induce unconscious feelings of gratitude and reciprocity. Gifts to physicians can perpetuate a mindset of entitlement.^14^

## INDIRECT CONFLICTS OF INTEREST

Incentives that are not directly financial but have financial implications such as career advancement may also represent potent COIs.^15^ Academic COIs may contribute to the disturbing prevalence of research irreproducibility.^15^

Preset convictions can cause investigators to overlook or selectively interpret data. The investigator who is so certain that the concept is correct may, even subconsciously, alter the eligibility criteria, or the number of subjects, to fit the data to the hypothesis, and reach the desired level of significance, a practice known as *p* hacking.^16^ A *t* test may be conducted on a tiny number of patients using data that are not normally distributed. The sample size may be kept small to ensure that adverse outcomes do not reach statistical significance.

## INDUSTRY PAYMENTS TO PROFESSIONAL SOCIETIES AND JOURNALS

Many professional societies, including plastic surgery societies, accept large payments from industry (ie, >$1 million annually). A key recommendation of a 2009 consensus report was a preliminary reduction in industry support to <25% of the operating budget of the professional medical association, with an ultimate goal of complete freedom from industry funding.^17^

Companies partner with our societies and fund journal supplements, compromising the separation of science and advertising. Industry involvement may extend to writing the manuscript, called “writing support.” Many medical journals derive a substantial proportion of their operating revenue from advertising.^18^ Sponsored supplements are typically written to support the marketing goals of the sponsor,^18^ and lack counterpoint discussions written by nonconflicted plastic surgeons. Brand names are featured in the titles. Editors may argue that supplements are treated with the same degree of scrutiny as regular publications,^19^ but the potential for inappropriate influence cannot be excluded.

Publication bias is a well-known problem.^5^ Researchers who are consultants, hold stock options, or receive royalties from companies, are much more likely to report positive results.^5,20^ Not surprisingly, industry is notoriously reluctant to publish negative findings.^5,21^ Some trial protocols include a provision for review of the manuscript by the funder before submission for publication, and the right to delay or even veto its publication.^5,22^ The timeline is pertinent. Plastic surgeons reporting on new products (eg, breast implants, implantable mesh, cryolipolysis, radiofrequency) may hold changing, often increasing, ownership stakes in successive publications, begging the question, when was this investment decision made and did it influence the research?

## EXAMPLES OF CONFLICT OF INTEREST IN PLASTIC SURGERY

### Breast Implants

The promotion of shaped, textured breast implants reflects the *quid pro quo* between the industry and surgeons.^23^ The highly praised gummy bear implant was always an inferior product. Shaped implants malrotate in 42% of patients.^24^ These devices are firm, may have palpable edges, can cause double capsules and seromas^25^ and are much more costly than smooth round alternatives. Most importantly, textured implants are linked to breast implant-associated anaplastic large-cell lymphoma.^26^ Hidalgo and Weinstein^27^ and others^28,29^ report no aesthetic advantage. Yet for decades now, textured, shaped implants have been promoted as superior to less expensive alternatives. Hall-Findlay^30^ writes: “We listen to the manufacturer’s claims and then years later we find that we have been misled – both by the manufacturers themselves and by those surgeons who are burdened by a conflict of interest.”

A study of nano-textured and micro-textured breast implants was recently published in a corporate-funded journal supplement.^31^ The authors report a complication rate of 0.3%, with 1 hematoma, no cases of implant malposition, no pain, no rippling, no ruptures, no redness, and no capsular contractures among 4,103 breast augmentations. The reoperation rate was <1%. The lead author reported no COI regarding this study, but accepted a position on the company’s medical advisory board immediately after submitting the article.^31^

### Implantable Mesh

The problem is not limited to breast implants. The COI regarding acellular dermal matrix is well documented.^32^ A recent article advocating the off-label use of implantable mesh in breast surgery was published by 2 authors who have a financial stake in the company that manufactures the mesh.^33^ A third author is a paid consultant and speaker. Galatea Surgical, a subsidiary of Tepha Inc. (Lexington, Ma.) financed the study, including medical writing, and referenced supplemental publications.^33^ Galatea is a corporate sponsor of the American Society for Aesthetic Plastic Surgery.^34^ The authors report that 100% of participating surgeons preferred to use mesh in all patients and the 1-year result was satisfactory in 100% of women.^33^ Such publications encourage plastic surgeons to adopt commercially driven practice patterns. A recent Continuing Medical Education article suggests that mesh support represents a paradigm shift.^35^ However, a nonconflicted analysis of mesh, and of the dated internal bra concept, finds no advantage.^36^

### Radiofrequency Treatments

A recent corporate-funded supplemental article, written by surgeons who are also shareholders, claims that radiofrequency-assisted liposuction (BodyTite, InMode Corp., Toronto, Canada) provides effective soft-tissue contraction, even creating an “internal brachioplasty scar.”^37^ This claim is based on a greater reduction in linear measurements than area measurements after radiofrequency-assisted liposuction compared with standard liposuction, a finding that is impossible to reconcile with basic geometry (the difference in area measurements must exceed linear changes).^38^ The authors offer a favorable return on investment analysis to justify the $205,000 purchase price, based on a $7,000 treatment fee.^37^

Conflicted author/investors frequently publish photographs that are not standardized in an effort to demonstrate a therapeutic benefit.^33,36,39,40^ A recent corporate-funded study on facial radiofrequency treatments with micro-needles was co-authored by a shareholder.^39^ The authors magnified the preoperative photograph of a nasolabial crease 58% to make it appear larger before treatment.^40^ Statistical errors included using a *t* test to compare nonparametric data, citing a *P* value of 1.00 for a comparison of nonidentical data, and a maximum range within 1 SD of the mean.^40^

## DISCOUNTS TO INVESTIGATORS

New transparency regulations help to inform the public about payments made to physicians.^41^ Unfortunately, it is not difficult to sidestep such reporting requirements. A well-known investigator may be given a device (eg, an ultrasonic liposuction machine) at a heavily discounted price. A breast implant manufacturer may provide its researchers with complimentary or discounted implants. There are many ways to reimburse surgeons indirectly. These considerations are substitutes for reportable cash payments, and they undermine the integrity of our research.

## COMPANY OFFICERS

Investigators who are not only passive investors but company officers and shareholders^42^ have a financial obligation to the company. A fiduciary responsibility makes it impossible to remain objective.^43^

## CLEARANCE BY U.S. FOOD AND DRUG ADMINISTRATION

When a device receives clearance by the U.S. Food and Drug Administration, it is labeled with a stamp of authority that is reassuring to the public. This label also serves as a powerful marketing tool. Unfortunately, the approval process is not protected from commercial influence. For example, Coolsculpting (Allergan plc, Dublin, Ireland) gained Food and Drug Administration clearance for treatment of the thighs based on studies performed by investigators that received major financial reimbursement.^44^ The company itself was allowed to conduct vital ultrasound and photographic imaging.^44^ The lead investigator was at one time a Zeltiq Aesthetics Inc. (Pleasanton, Calif.) paid consultant and shareholder.^44^ Zeltiq was purchased in 2017 by Allergan plc (Dublin, Ireland) for $2.48 billion.^45^

## HONEST REPORTING

Corporate-funded studies consistently report unusually low complication rates, speedy recoveries, high rates of patient satisfaction, high “conversion rates,” and even the prospects for cross-selling.^46^ These sales-oriented characteristics undermine hard-won gains in honest reporting and the recognition of the importance of evidence-based medicine in our scientific journals.

Adoption of unsound treatments and devices based on biased studies can have harmful long-term ramifications through a “rippling effect.”^5^ Biased studies may be referenced in practice guidelines.^5^ Physician disillusionment, especially after the purchase of an expensive yet underperforming device, may be a factor in physician burnout.

## CONSULTANTS

Although physicians may consider themselves to be ethical professionals, many doctors remain unaware of the subconscious bias that industry relationships create.^47^ The practice of doctors accepting payments from companies has gone on for decades without a critical review. Are plastic surgeons truly acting as consultants, or is “consultant” a euphemism for receipt of a payment to shape one’s opinion in favor of the product and confer loyalty? Industry payments, which may be viewed as kickbacks, have created serious legal difficulties for physicians.^2^ Lopez et al.^20^ found that self-reported COIs have declined in recent years, but the proportion of consultantships has increased.

In proposing an end to industry influence and regaining the public trust, a committee formed by the Institute of Medicine finds that continuing medical education “has become far too reliant on industry funding,” which “tends to promote a narrow focus on products.”^48^ The committee recommends restricting consultantships to the provision of objective technical advice paid at fair market value, documented in written contracts. Moreover, companies “should not involve physicians and patients in marketing projects that are presented as clinical research.”^48^

## RECOMMENDATIONS

The International Committee of Medical Journal Editors disclosure form insists that contributors disclose relevant financial relationships.^49^ In 2010, the Council of Medical Specialty Societies published a code for interactions with companies, with a provision that prohibits society officers and journal editors from accepting any compensation from industry.^50^

To facilitate transparency of disclosure, Congress passed the Physician Payments Sunshine Act, which requires commercial companies to report any “transfer of value” to any physician, with a $10 threshold.^51^ In October 2010, ProPublica introduced Dollars for Docs, a central search engine for physician payments.^41^

Luce^6^ proposes that plastic surgeons with conflicts be excused as manuscript discussants and reviewers. Lichter^50^ recommends that a presentation with a COI should be balanced by a nonconflicted counterpoint discussant. The American Society of Plastic Surgeons has adopted a requirement for disclosure of financial conflicts in dollar ranges (ie, $100–$1,000, $1,001–$5,000, $5,001–$10,000, etc.).^2^ These are reasonable first steps.

Device evaluation does not necessarily require industry funding, as evidenced by the research efforts of investigators without financial conflicts.^25,27^ Publication of independent research in a highly respected peer-reviewed journal and the accolades that come with it provide more than adequate compensation, and potential for practice building and career advancement.

It is impossible to reconcile corporate sponsorship with unbiased research. Physician investigators should consider declining any paid consultancies and all forms of indirect corporate reimbursement. Study design and implementation, and manuscript preparation should not be outsourced.

## RELINQUISHING INDUSTRY FINANCIAL SUPPORT

Asking attendees to visit the exhibits, “without which none of this [i.e., the meeting] would be possible” is a familiar refrain at meetings. The physician-industry complex has gone on for so long that plastic surgeons may find it difficult to imagine an arms-length relationship. Without industry sponsorship, plastic surgeons can expect to pay more to attend meetings and Continuing Medical Education activities, but the prices of devices and implants are likely to fall as companies are relieved of the tremendous financial burden^6^ of payments to physicians and societies. The net overall financial effect to physicians is zero, but medical integrity is restored. Relinquishing industry financial support represents a bold step, but recent examples of the influence of financial conflicts underscore the magnitude of the problem.

As reported by Rohrich et al.,^52^ when Goldwyn stepped down as the longtime former editor of *Plastic and Reconstructive Surgery*, he worried most about commercial influence and keeping the specialty “pure.” He cautioned the incoming managing editor that he would need a strong sense of ethics because “you’ll need them in this business.” Goldwyn,^53^ quoting his father, wrote: “It is amazing how easy it is to be truthful if one wants to be.”

## CONCLUSIONS

It is impossible for investigators to function as highly paid consultants and remain unbiased. Disclosure, including the amount of money paid, allows the audience to determine the importance of the conflict. Separation of commerce and science in our journal publications is vital so that scientific publications do not become marketing tools. Plastic surgeons must be better advocates for our patients and their pocketbooks. Part of the privilege of caring for patients is to be mindful of their finances and their health.^14^ Most importantly, our societies need to reconsider corporate partnership. Editors are already aware of the professional positions of reviewers and the need to protect the article that upsets the apple cart. As Descartes famously observed, “doubt is the origin of wisdom.”^54^ Progress is only made possible by challenging the status quo.

## References

[1] Conflict of interest. JAMA. 2017;317:1705 1812. 10.1001/jama.2017.4044 28464142

[2] Snyder P Society continues push toward COI transparency. Plast Surg News. 2018:18 19.

[3] Tringale KR Marshall D Mackey TK Types and distribution of payments from industry to physicians in 2015. JAMA. 2017;317:1774 1784. 28464140 10.1001/jama.2017.3091 PMC5470350

[4] Luce EA Jackman CA Disclosure of financial conflicts of interest in plastic and reconstructive surgery. Plast Reconstr Surg. 2017;140:635 639. 28841626 10.1097/PRS.0000000000003598

[5] Lopez J Lopez S Means J Financial conflicts of interest: an association between funding and findings in plastic surgery. Plast Reconstr Surg. 2015;136:690e 697e. 10.1097/PRS.0000000000001718 26505726

[6] Luce EA Financial conflicts of interest in plastic surgery: background, potential for bias, disclosure, and transparency. Plast Reconstr Surg. 2015;135:1149 1155. 25285680 10.1097/PRS.0000000000000788

[7] Pizzo PA Lawley TJ Rubenstein AH Role of leaders in fostering meaningful collaborations between academic medical centers and industry while also managing individual and institutional conflicts of interest. JAMA. 2017;317:1729 1730. 28464157 10.1001/jama.2017.2573

[8] Thornton JP Conflict of interest and legal issues for investigators and authors. JAMA. 2017;317:1761 1762. 28464169 10.1001/jama.2017.4235

[9] Fineberg HV Conflict of interest: why does it matter? JAMA. 2017;317:1717 1718. 28464149 10.1001/jama.2017.1869

[10] McCoy MS Emanuel EJ Why there are no “potential” conflicts of interest. JAMA. 2017;317:1721 1722. 28464154 10.1001/jama.2017.2308

[11] Lo B Grady D Payments to physicians: does the amount of money make a difference? JAMA. 2017;317:1719 1720. 28464150 10.1001/jama.2017.1872

[12] DeJong C Aguilar T Tseng CW Pharmaceutical industry-sponsored meals and physician prescribing patterns for Medicare beneficiaries. JAMA Intern Med. 2016;176:1114 1122. 27322350 10.1001/jamainternmed.2016.2765

[13] Ornstein C Public disclosure of payments to physicians from industry. JAMA. 2017;317:1749 1750. 28464158 10.1001/jama.2017.2613

[14] Steinbrook R Physicians, industry payments for food and beverages, and drug prescribing. JAMA. 2017;317:1753 1754. 28464155 10.1001/jama.2017.2477

[15] Flier JS Conflict of interest among medical school faculty: achieving a coherent and objective approach. JAMA. 2017;317:1731 1732. 28464148 10.1001/jama.2017.1751

[16] Head ML Holman L Lanfear R The extent and consequences of p-hacking in science. PLoS Biol. 2015;13:e1002106. 25768323 10.1371/journal.pbio.1002106 PMC4359000

[17] Rothman DJ McDonald WJ Berkowitz CD Professional medical associations and their relationships with industry: a proposal for controlling conflict of interest. JAMA. 2009;301:1367 1372. 19336712 10.1001/jama.2009.407

[18] Nissen SE Conflicts of interest and professional medical associations: progress and remaining challenges. JAMA. 2017;317:1737 1738. 28464156 10.1001/jama.2017.2516

[19] Nahai F Disclosing conflicts of interest to maintain ethical integrity. Aesthet Surg J. 2011;31:591 593. 21719871 10.1177/1090820X11412525

[20] Lopez J Musavi L Quan A Trends, frequency, and nature of surgeon-reported conflicts of interest in plastic surgery. Plast Reconstr Surg. 2017;140:852 861. 28953741 10.1097/PRS.0000000000003683

[21] Dickersin K Rennie D Registering clinical trials. JAMA. 2003;290:516 523. 12876095 10.1001/jama.290.4.516

[22] Fontanarosa P Bauchner H Conflict of interest and medical journals. JAMA. 2017;317:1768 1771. 28464125 10.1001/jama.2017.4563

[23] Swanson E Textured breast implants, anaplastic large-cell lymphoma (ALCL), and conflict of interest. Plast Reconstr Surg. 2017;139:558e 559e. 10.1097/PRS.0000000000002966 28125539

[24] Sieber DA Stark RY Chase S Clinical evaluation of shaped gel breast implant rotation using high-resolution ultrasound. Aesthet Surg J. 2017;37:290 296. 28207033 10.1093/asj/sjw179

[25] Hall-Findlay EJ Breast implant complication review: double capsules and late seromas. Plast Reconstr Surg. 2011;127:56 66. 21200201 10.1097/PRS.0b013e3181fad34d

[26] Brody GS Deapen D Taylor CR Anaplastic large cell lymphoma occurring in women with breast implants: analysis of 173 cases. Plast Reconstr Surg. 2015;135:695 705. 25490535 10.1097/PRS.0000000000001033

[27] Hidalgo DA Weinstein AL Intraoperative comparison of anatomical versus round implants in breast augmentation: a randomized controlled trial. Plast Reconstr Surg. 2017;139:587 596. 28234826 10.1097/PRS.0000000000003114

[28] Friedman T Davidovitch N Scheflan M Comparative double blind clinical study on round versus shaped cohesive gel implants. Aesthet Surg J. 2006;26:530 536. 19338941 10.1016/j.asj.2006.08.004

[29] Gahm J Edsander-Nord A Jurell G No differences in aesthetic outcome or patient satisfaction between anatomically shaped and round expandable implants in bilateral breast reconstructions: a randomized study. Plast Reconstr Surg. 2010;126:1419 1427. 20639801 10.1097/PRS.0b013e3181ef8b01

[30] Hall-Findlay EJ Discussion: late seromas and breast implants: theory and practice. Plast Reconstr Surg. 2012;130:436 438. 22842415 10.1097/PRS.0b013e31825910cb

[31] Sforza M Zaccheddu R Alleruzzo A Preliminary 3-year evaluation of experience with SilkSurface and VelvetSurface Motiva Silicone Breast Implants: a single-center experience with 5813 consecutive breast augmentation cases. Aesthet Surg J. 2018;38:S62 S73. 29040364 10.1093/asj/sjx150 PMC5952962

[32] Lopez J Prifogle E Nyame TT The impact of conflicts of interest in plastic surgery: an analysis of acellular dermal matrix, implant-based breast reconstruction. Plast Reconstr Surg. 2014;133:1328 1334. 24867714 10.1097/PRS.0000000000000172

[33] Adams WP Jr Baxter R Glicksman C The use of poly-4-hydroxybutyrate (P4HB) scaffold in the ptotic breast: a multicenter clinical study. Aesthet Surg J. 2018;38:502 518. 29401215 10.1093/asj/sjy022

[34] ASAPS Corporate Support Program. Available at https://www.surgery.org/professionals/corporate-support-program. Accessed June 3, 2018.

[35] Qureshi AA Myckatyn TM Tenenbaum MM Mastopexy and mastopexy-augmentation. Aesthet Surg J. 2018;38:374 384. 29365038 10.1093/asj/sjx181

[36] Swanson E Evaluating the effect of implantable mesh in mammaplasty. Aesthet Surg J. 2018;38:NP103 NP105. 10.1093/asj/sjy056 29701754

[37] Theodorou SJ Del Vecchio D Chia CT Soft tissue contraction in body contouring with radiofrequency-assisted liposuction: a treatment gap solution. Aesthet Surg J. 2018;38:S74 S83. 29767716 10.1093/asj/sjy037

[38] Swanson E Does radiofrequency assistance improve skin contraction after liposuction? Plast Reconstr Surg Glob Open. 2015;3:e545. 26579351 10.1097/GOX.0000000000000531 PMC4634182

[39] Samadi A Nasrollahi SA Janani L Combination of fractional radiofrequency and thermo-contraction systems for facial skin rejuvenation: a clinical and histological study. Aesthet Surg. J. Published online June 20, 2018. 10.1093/asj/sjy152 29931298

[40] Swanson E A critique of radiofrequency treatments for facial rejuvenation. Aesthet Surg J. Accepted July 2018. 10.1093/asj/sjy192 30608573

[41] Dollars for Docs website. Available at http://www.propublica.org/docdollars/. Accessed June 3, 2018.

[42] Lim AF Weintraub J Kaplan EN The embrace device significantly decreases scarring following scar revision surgery in a randomized controlled trial. Plast Reconstr Surg. 2014;133:398 405. 24105084 10.1097/01.prs.0000436526.64046.d0 PMC4874339

[43] Swanson E Tension shielding with the embrace device: does it really improve scars? Plast Reconstr Surg. 2014;134:662e 664e. 10.1097/PRS.0000000000000568 25357067

[44] Stevens WG Bachelor EP Cryolipolysis conformable-surface applicator for nonsurgical fat reduction in lateral thighs. Aesthet Surg J. 2015;35:66 71. 25568236 10.1093/asj/sju024 PMC4462597

[45] Allergan will buy Zeltiq for $2.48 billion. Available at http://fortune.com/2017/02/13/allergan-zeltiq-acquisition/. Accessed June 3, 2018.

[46] Swanson E Cryolipolysis: the importance of scientific evaluation of a new technique. Aesthet Surg J. 2015;35:NP116 NP119. 25829438 10.1093/asj/sju069

[47] Chimonas S Brennan TA Rothman DJ Physicians and drug representatives: exploring the dynamics of the relationship. J Gen Intern Med. 2007;22:184 190. 17356984 10.1007/s11606-006-0041-z PMC1824740

[48] Steinbrook R Controlling conflict of interest—proposals from the Institute of Medicine. N Engl J Med. 2009;360:2160 2163. 19403898 10.1056/NEJMp0810200

[49] International Committee of Medical Journal Editors. Author responsibilities–conflicts of interest. Recommendations for the conduct, reporting, editing, and publication of scholarly work in medical journals. Available at http://www.icmje.org/recommendations. Accessed June 18, 2018. 25558501

[50] Lichter AS Conflict of interest and the integrity of the medical profession. JAMA. 2017;317:1725 1726. 28464163 10.1001/jama.2017.3191

[51] Centers for Medicare & Medicaid services. Available at https://www.cms.gov/openpayments/. Accessed June 17, 2018.

[52] Rohrich RJ Wills A Mulligan JR To have some friends: a tribute to Robert Goldwyn, M.D., 1930 to 2010—Plastic and Reconstructive Surgery editor emeritus dies at age 79. Plast Reconstr Surg. 2010;126:691 695. 20712077 10.1097/prs.0b013e3181e5049c

[53] Goldwyn RM Wanted: real clinical results. Plast Reconstr Surg. 2004;114:1000 1001.

[54] Descartes quote. Available at https://www.goodreads.com/quotes/247631-doubt-is-the-origin-of-wisdom. Accessed June 3, 2018.

